# Supplementary material for: Time trajectories in the transcriptomic response to exercise - a meta-analysis
Source: Nat Commun. 2021 Jun 9;12:3471. doi: 10.1038/s41467-021-23579-x (PMC8190306; doi:10.1038/s41467-021-23579-x)
Supplement: Supplementary file 1 — Supplementary Information [file 41467_2021_23579_MOESM1_ESM.pdf]

Time trajectories in the transcriptomic response to exercise - a meta-analysis

Supplementary Figures

Acute							
Muscle				Blood			
Pathway	Gene ranks	NES	Adj P	Pathway	Gene ranks	NES	Adj P
Degradation of the extracellular matrix		1.94	4.5x10^{-3}	Attenuation phase		2.15	3.9x10^{-3}
Extracellular matrix organization		1.97	4.5x10^{-3}	Cellular response to heat stress		1.98	3.9x10^{-3}
Interleukin-4 and Interleukin-13 signaling		2.35	4.5x10^{-3}	ESR-mediated signaling		1.72	3.9x10^{-3}
Complex I biogenesis		-2.49	9x10^{-3}	Nucleobase catabolism		-2.23	1.1x10^{-2}
Mitochondrial translation		-2.17	9.x10^{-3}	Erythrocytes take up carbon dioxide and release oxygen		-2.26	1.8x10^{-2}
Mitochondrial translation elongation		-2.20	9x10^{-3}	Metabolism of nucleotides		-2.18	1.8x10^{-2}
**Long-term**							
Muscle				Blood			
Pathway	Gene ranks	NES	Adj P	Pathway	Gene ranks	NES	Adj P
Assembly of collagen fibrils and other multimeric structures		2.48	1.2x10^{-3}	Activation of the mRNA upon binding of the cap-binding complex and eIFs		2.90	1.3x10^{-3}
Citric acid cycle (TCA cycle)		2.11	1.2x10^{-3}	Cap-dependent Translation Initiation		3.24	1.3x10^{-3}
Collagen biosynthesis and modifying enzymes		2.39	1.2x10^{-3}	DNA Replication Pre-Initiation		2.05	1.3x10^{-3}
Activation of the mRNA upon binding of the cap-binding complex and eIFs		-2.11	1.2x10^{-3}	Antimicrobial peptides		-2.36	1.3x10^{-3}
Cap-dependent Translation Initiation		-2.32	1.2x10^{-3}	Extracellular matrix organization		-2.06	1.3x10^{-3}
Cardiac conduction		-1.88	1.2x10^{-3}	Phospholipid metabolism		-1.88	1.3x10^{-3}

Supplementary Figure 1. Top GSEA results. In each analysis the top three up- and down-regulated pathways are shown.

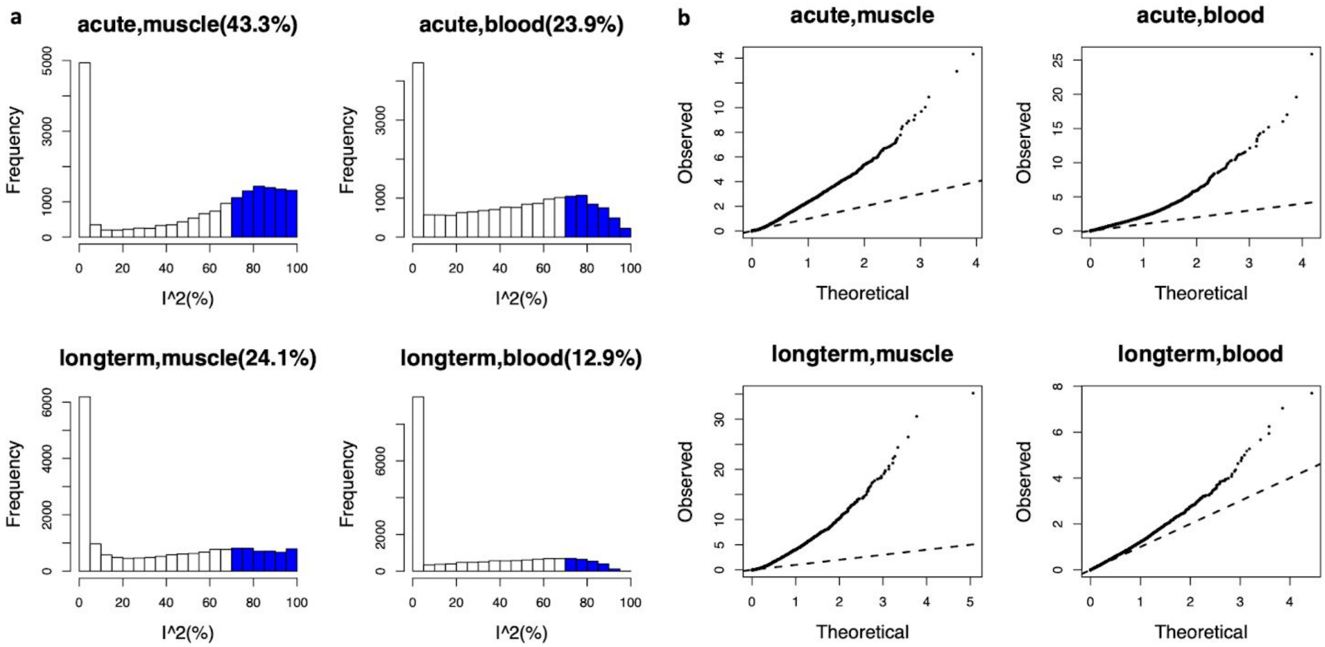

Supplementary Figure 2. Statistics of the naive random effects meta-analysis. For each gene in each of the four analysis types we computed the true heterogeneity I^2 score and the model's p-value. A) Histograms of the I^2 scores. The blue region represents genes with $I^2 > 70\%$. B) QQ-plots illustrate how the observed p-value distributions are inflated with low p-values.

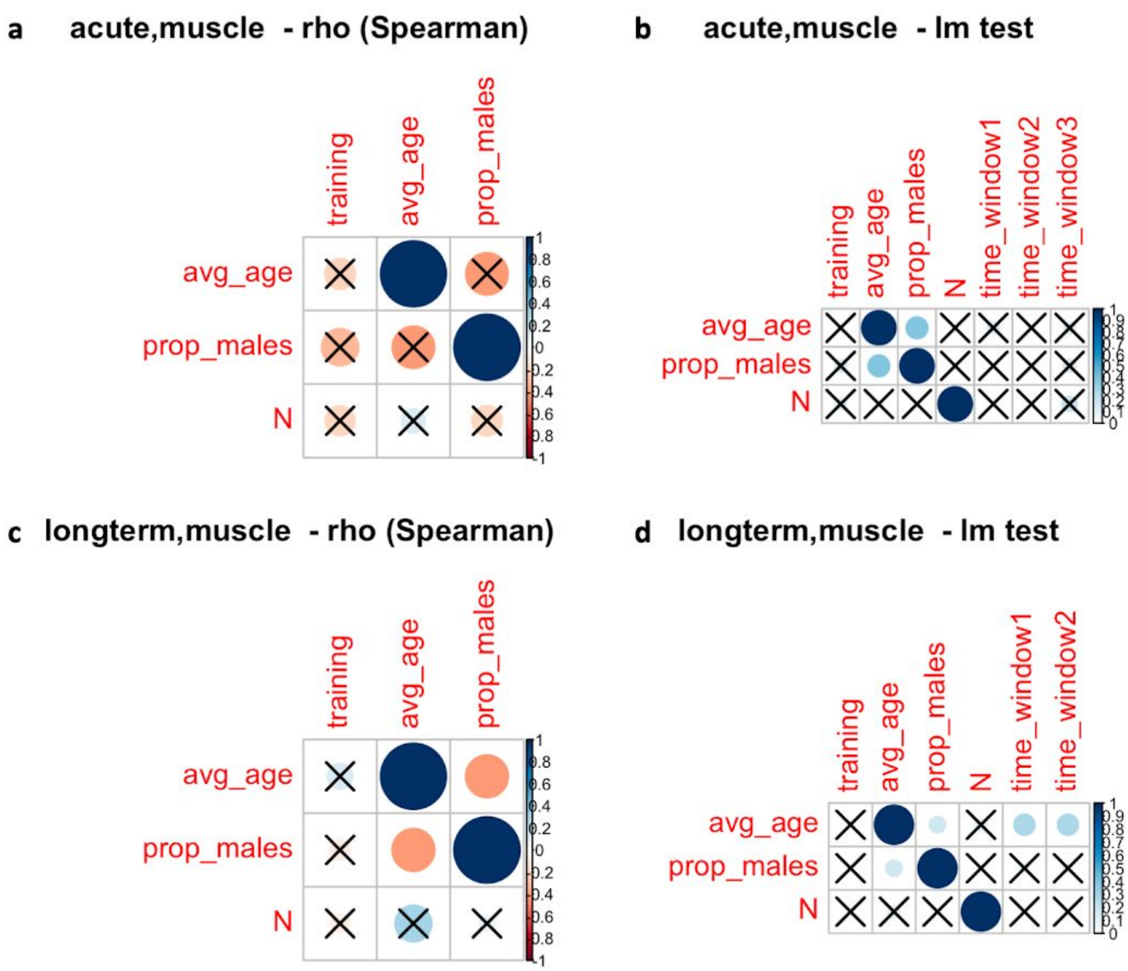

Supplementary Figure 3. Spearman correlations of moderators (covariates) across the muscle cohorts. N is the number of subjects. For time, acute studies were binned into three windows (1:0-1h, 2: 2-6h; 3:20h+). Long-term studies were binned into two time windows (up to 150 days or greater). X indicates that the correlation between a pair of moderators is not significant. Circle size is proportional to either the Spearman correlation coefficient (left) or the R^2 score of the linear regression analysis (right).

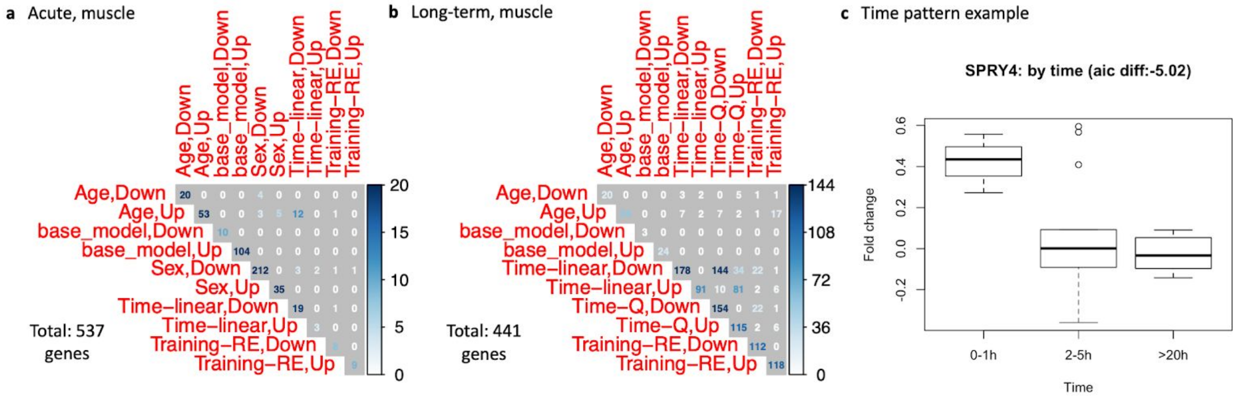

Supplementary Figure 4. Selected models and their moderators (covariates). **a-b** The matrices show the co-occurrence of covariates in selected models. Each number represents the number of genes whose models had the two moderators. The diagonal shows the total number of models for each moderator. Sex: the proportion of males in a study. Time-linear and Time-Q are orthogonal polynomials used to model the time response (e.g., a linear trend will approximate a monotone up- or down-regulation response). Training-RE: a binary covariate specifying if the cohort included resistance training. Up/Down in the covariate name specifies if the regression coefficient is positive or negative, respectfully. **c** *SPRY4* in acute muscle cohorts is up-regulated only in the first time window. Each boxplot represents the distribution of fold changes in a given time window. In addition, each boxplot shows the median, and first and third quartiles. The whiskers extend from the hinge to the largest and lowest values, but no further than 1.5 * (the inter-quantile range). In this case the selected model had time associated features that represent both a linear and a quadratic trend.

SMAD3

Cohort	N	Type	Age	%M	Time
A_11	18	EE	52.1	100	0-1h
A_26	17	EE	27	100	0-1h
A_33	28	EE	33	64	0-1h
A_1	18	EE	23.9	100	2-5h
A_1	18	EE	23.9	100	2-5h
A_2	18	RE	23.9	100	2-5h
A_2	18	RE	23.9	100	2-5h
A_6	24	EE	32.8	100	2-5h
A_7	14	RE	23.5	57	2-5h
A_9	20	RE	47.8	0	2-5h
A_10	25	RE	47.8	0	2-5h
A_24	20	EE	22.6	100	2-5h
A_26	17	EE	27	100	2-5h
A_27	12	RE	27	100	2-5h
A_29	24	RE	21.4	100	2-5h
A_34	32	EE	25	100	2-5h
A_6	24	EE	32.8	100	>20h
A_18	19	RE	22	100	>20h
A_18	19	RE	22	100	>20h
A_25	20	RE	26	100	>20h
A_29	24	RE	21.4	100	>20h
A_34	32	EE	25	100	>20h
A_34	32	EE	25	100	>20h

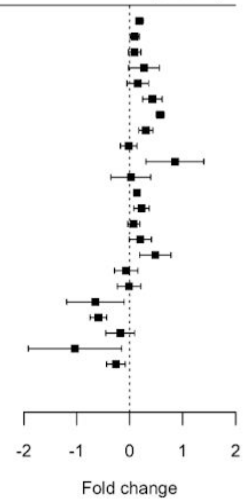

NR4A1

Cohort	N	Type	Age	%M	Time
A_11	18	EE	52.1	100	0-1h
A_26	17	EE	27	100	0-1h
A_33	28	EE	33	64	0-1h
A_1	18	EE	23.9	100	2-5h
A_1	18	EE	23.9	100	2-5h
A_2	18	RE	23.9	100	2-5h
A_2	18	RE	23.9	100	2-5h
A_6	24	EE	32.8	100	2-5h
A_7	14	RE	23.5	57	2-5h
A_9	20	RE	47.8	0	2-5h
A_10	25	RE	47.8	0	2-5h
A_24	20	EE	22.6	100	2-5h
A_26	17	EE	27	100	2-5h
A_27	12	RE	27	100	2-5h
A_29	24	RE	21.4	100	2-5h
A_34	32	EE	25	100	2-5h
A_6	24	EE	32.8	100	>20h
A_18	19	RE	22	100	>20h
A_18	19	RE	22	100	>20h
A_25	20	RE	26	100	>20h
A_29	24	RE	21.4	100	>20h
A_34	32	EE	25	100	>20h
A_34	32	EE	25	100	>20h

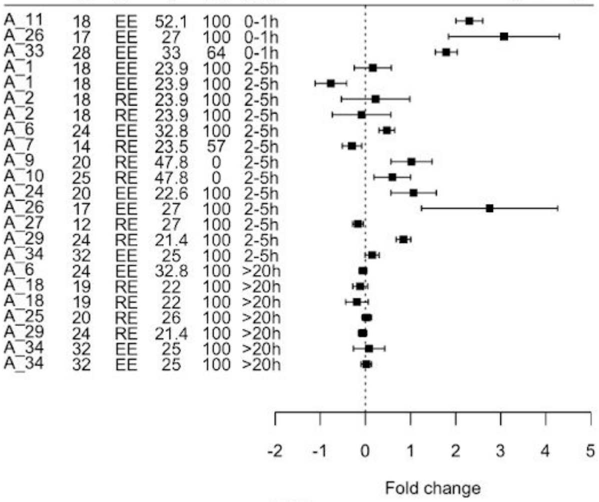

HES1

Cohort	N	Type	Age	%M	Time
A_11	18	EE	52.1	100	0-1h
A_26	17	EE	27	100	0-1h
A_33	28	EE	33	64	0-1h
A_1	18	EE	23.9	100	2-5h
A_1	18	EE	23.9	100	2-5h
A_2	18	RE	23.9	100	2-5h
A_2	18	RE	23.9	100	2-5h
A_6	24	EE	32.8	100	2-5h
A_7	14	RE	23.5	57	2-5h
A_9	20	RE	47.8	0	2-5h
A_10	25	RE	47.8	0	2-5h
A_24	20	EE	22.6	100	2-5h
A_26	17	EE	27	100	2-5h
A_27	12	RE	27	100	2-5h
A_29	24	RE	21.4	100	2-5h
A_34	32	EE	25	100	2-5h
A_6	24	EE	32.8	100	>20h
A_18	19	RE	22	100	>20h
A_18	19	RE	22	100	>20h
A_25	20	RE	26	100	>20h
A_29	24	RE	21.4	100	>20h
A_34	32	EE	25	100	>20h
A_34	32	EE	25	100	>20h

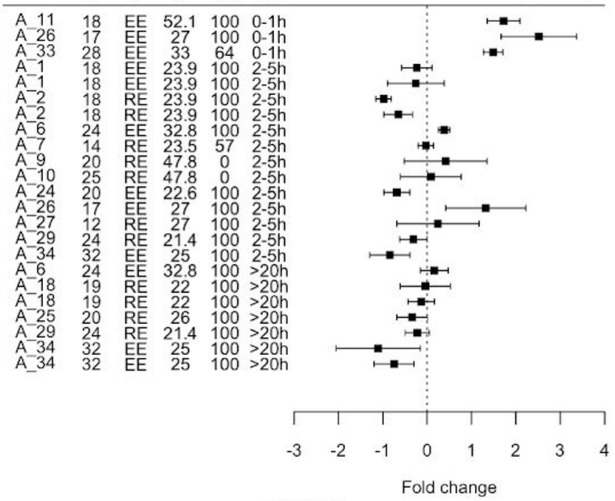

ID1

Cohort	N	Type	Age	%M	Time
A_11	18	EE	52.1	100	0-1h
A_26	17	EE	27	100	0-1h
A_33	28	EE	33	64	0-1h
A_1	18	EE	23.9	100	2-5h
A_1	18	EE	23.9	100	2-5h
A_2	18	RE	23.9	100	2-5h
A_2	18	RE	23.9	100	2-5h
A_6	24	EE	32.8	100	2-5h
A_7	14	RE	23.5	57	2-5h
A_9	20	RE	47.8	0	2-5h
A_10	25	RE	47.8	0	2-5h
A_24	20	EE	22.6	100	2-5h
A_26	17	EE	27	100	2-5h
A_27	12	RE	27	100	2-5h
A_29	24	RE	21.4	100	2-5h
A_34	32	EE	25	100	2-5h
A_6	24	EE	32.8	100	>20h
A_18	19	RE	22	100	>20h
A_18	19	RE	22	100	>20h
A_25	20	RE	26	100	>20h
A_29	24	RE	21.4	100	>20h
A_34	32	EE	25	100	>20h
A_34	32	EE	25	100	>20h

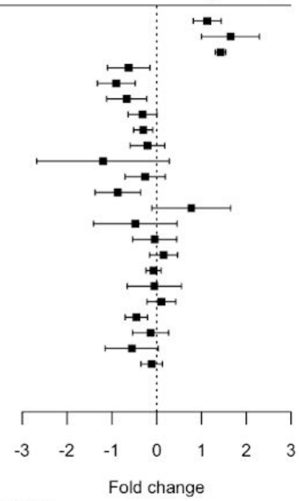

SCN2B

Cohort	N	Type	Age	%M	Time
A_11	18	EE	52.1	100	0-1h
A_26	17	EE	27	100	0-1h
A_33	28	EE	33	64	0-1h
A_1	18	EE	23.9	100	2-5h
A_1	18	EE	23.9	100	2-5h
A_2	18	RE	23.9	100	2-5h
A_2	18	RE	23.9	100	2-5h
A_6	24	EE	32.8	100	2-5h
A_7	14	RE	23.5	57	2-5h
A_9	20	RE	47.8	0	2-5h
A_10	25	RE	47.8	0	2-5h
A_24	20	EE	22.6	100	2-5h
A_26	17	EE	27	100	2-5h
A_27	12	RE	27	100	2-5h
A_29	24	RE	21.4	100	2-5h
A_34	32	EE	25	100	2-5h
A_6	24	EE	32.8	100	>20h
A_18	19	RE	22	100	>20h
A_18	19	RE	22	100	>20h
A_25	20	RE	26	100	>20h
A_29	24	RE	21.4	100	>20h
A_34	32	EE	25	100	>20h
A_34	32	EE	25	100	>20h

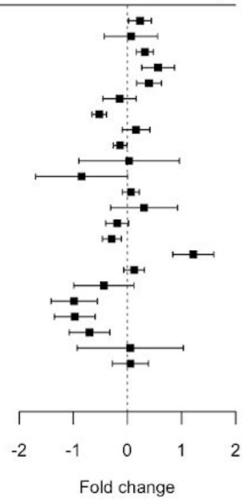

SLC25A25

Cohort	N	Type	Age	%M	Time
A_11	18	EE	52.1	100	0-1h
A_26	17	EE	27	100	0-1h
A_33	28	EE	33	64	0-1h
A_1	18	EE	23.9	100	2-5h
A_1	18	EE	23.9	100	2-5h
A_2	18	RE	23.9	100	2-5h
A_2	18	RE	23.9	100	2-5h
A_6	24	EE	32.8	100	2-5h
A_7	14	RE	23.5	57	2-5h
A_9	20	RE	47.8	0	2-5h
A_10	25	RE	47.8	0	2-5h
A_24	20	EE	22.6	100	2-5h
A_26	17	EE	27	100	2-5h
A_27	12	RE	27	100	2-5h
A_29	24	RE	21.4	100	2-5h
A_34	32	EE	25	100	2-5h
A_6	24	EE	32.8	100	>20h
A_18	19	RE	22	100	>20h
A_18	19	RE	22	100	>20h
A_25	20	RE	26	100	>20h
A_29	24	RE	21.4	100	>20h
A_34	32	EE	25	100	>20h
A_34	32	EE	25	100	>20h

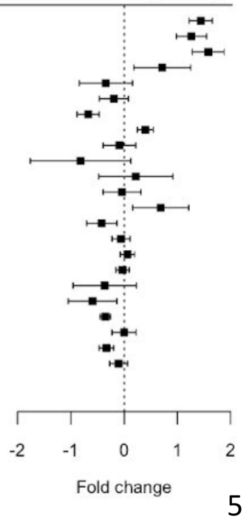

Supplementary Figure 5. Forest plots for genes selected for validation from the skeletal muscle acute exercise meta-analysis. In each forest plot rows represent the 95% confidence interval of a fold change of a cohort in a given time point. Thus, in each interval the center represents the fold change estimate and the error bars are proportional to the fold change standard error.

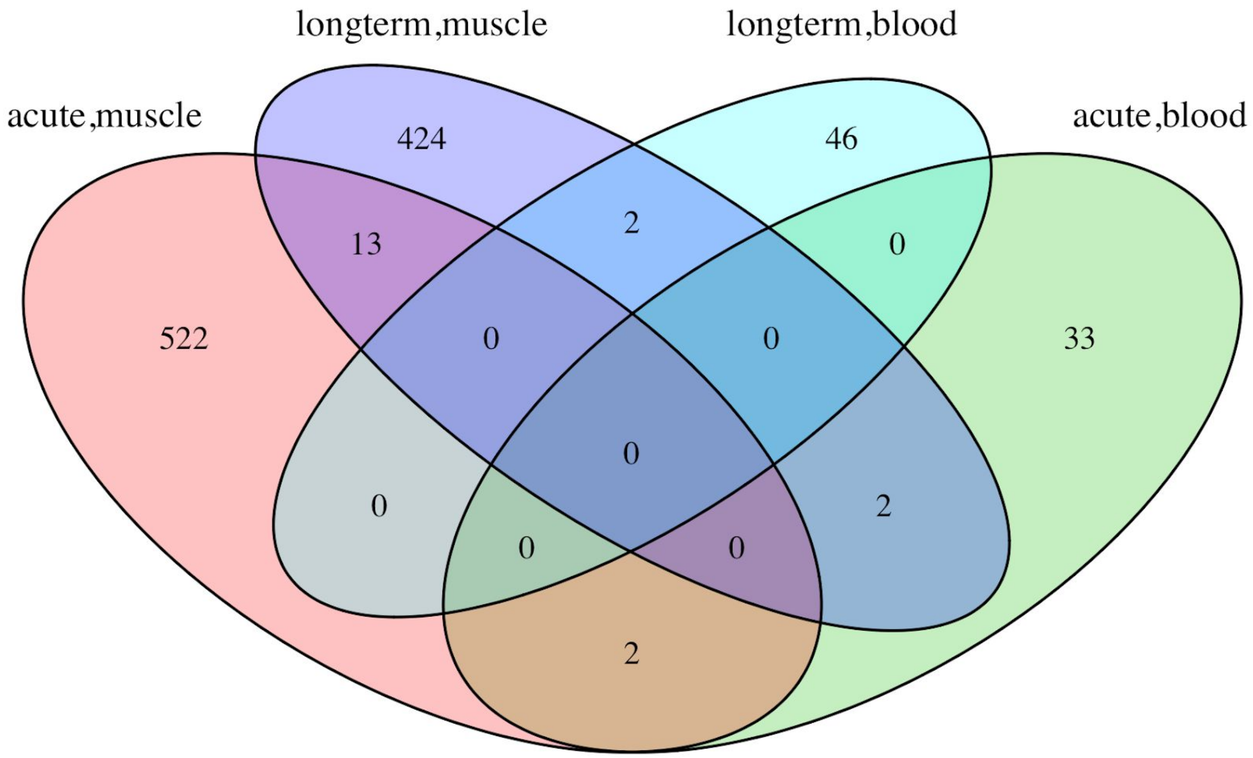

Supplementary Figure 6. The overlap between the gene sets discovered in each of the four meta-analyses. Pink – acute muscle changes, purple – long-term muscle changes, turquoise – long-term blood changes, green – acute blood changes.

Supplementary Tables

Supplementary Table 1. Primer sequences used for qRT-PCR experiments.

Primer_Name	Forward_primer	Reverese_primer
HES1	GCCTATTATGGAGAAAAGACG	CTATCTTTCTTCAGAGCATCC
ID1	ACTAGTCACCAGAGACTTTAG	AAATCTGAGAAGCACCAAAC
GAPDH	CCTCCTGCACCACCAACTGCTT	GAGGGGGCACCACCAGTCTT T
MTMR3	ATCAGGTGTCAGTTTTCAAC	TCTCCATGTTGCTCTTTTTC
NR4A1	AGAAAAACGCCAAGTACATC	GTTCGGACAACTTCCTTC
RPS18	CTTCCACAGGAGGCCTACAC	CCATCGATGTTGGTGTTGAG
SCN2B	CTACACAGTGAACCACAAAC	CAGCTTCAGGTTAATGATCTTC
SLC25A25	GATCATGAGAAGAAGCTGAG	CTGTTCAGATATCTTGACTCC
SMAD3	CTACCAGAGAGTAGAGACAC	TCTCTGGAATATTGCTCTGG
