# Supplementary material for: Time trajectories in the transcriptomic response to exercise - a meta-analysis
Source: Nat Commun. 2021 Jun 9;12:3471. doi: 10.1038/s41467-021-23579-x (PMC8190306; doi:10.1038/s41467-021-23579-x)
Supplement: Supplementary file 13 — Description of Additional Supplementary Files [file 41467_2021_23579_MOESM13_ESM.pdf]

Title: Supplementary Data 1.
Description: Cohort metadata and moderators.

Title: Supplementary Data 2.
Description: Full curated acute exercise transcriptome dataset annotations.

Title: Supplementary Data 3.
Description: Full curated long-term training transcriptome dataset annotations.

Title: Supplementary Data 4.
Description: GSEA results. Each row represents an enriched pathway in one of the four meta-analyses, enriched at 10% FDR.

Title: Supplementary Data 5.
Description: Model selection results. Each row represents a significant (meta-analysis,gene, moderator) triplet. The model's AICc difference and significance are shown. In addition, the gene clustering results are presented.

Title: Supplementary Data 6.
Description: Model selection results in acute muscle: full models. Each row represents a (gene, moderator/intercept) pair. The effect size and p-value are shown for each pair.

Title: Supplementary Data 7.
Description: Model selection results in acute blood: full models. Each row represents a (gene, moderator/intercept) pair. The effect size and p-value are shown for each pair.

Title: Supplementary Data 8.
Description: Model selection results in long-term muscle: full models. Each row represents a (gene, moderator/intercept) pair. The effect size and p-value are shown for each pair.

Title: Supplementary Data 9.
Description: Model selection results in long-term, blood: full models. Each row represents a (gene, moderator/intercept) pair. The effect size and p-value are shown for each pair.

Title: Supplementary Data 10.
Description: GO enrichment analysis results.

Title: Supplementary Data 11.
Description: Reactome pathway enrichment analysis results.
