# Supplementary material for: Time trajectories in the transcriptomic response to exercise - a meta-analysis
Source: Nat Commun. 2021 Jun 9;12:3471. doi: 10.1038/s41467-021-23579-x (PMC8190306; doi:10.1038/s41467-021-23579-x)
Supplement: Supplementary file 15 — Reporting Summary [file 41467_2021_23579_MOESM15_ESM.pdf]

nature research

Corresponding author(s): Euan A. Ashley

Last updated by author(s): Dec 1, 2020

Reporting Summary

Nature Research wishes to improve the reproducibility of the work that we publish. This form provides structure for consistency and transparency in reporting. For further information on Nature Research policies, see our Editorial Policies and the Editorial Policy Checklist.

Statistics

For all statistical analyses, confirm that the following items are present in the figure legend, table legend, main text, or Methods section.

n/a	Confirmed	
☐	☒	The exact sample size (*n*) for each experimental group/condition, given as a discrete number and unit of measurement
☐	☒	A statement on whether measurements were taken from distinct samples or whether the same sample was measured repeatedly
☐	☒	The statistical test(s) used AND whether they are one- or two-sided<br>*Only common tests should be described solely by name; describe more complex techniques in the Methods section.*
☐	☒	A description of all covariates tested
☐	☒	A description of any assumptions or corrections, such as tests of normality and adjustment for multiple comparisons
☐	☒	A full description of the statistical parameters including central tendency (e.g. means) or other basic estimates (e.g. regression coefficient) AND variation (e.g. standard deviation) or associated estimates of uncertainty (e.g. confidence intervals)
☐	☒	For null hypothesis testing, the test statistic (e.g. *F*, *t*, *r*) with confidence intervals, effect sizes, degrees of freedom and *P* value noted<br>*Give P values as exact values whenever suitable.*
☒	☐	For Bayesian analysis, information on the choice of priors and Markov chain Monte Carlo settings
☐	☒	For hierarchical and complex designs, identification of the appropriate level for tests and full reporting of outcomes
☐	☒	Estimates of effect sizes (e.g. Cohen's *d*, Pearson's *r*), indicating how they were calculated

Our web collection on statistics for biologists contains articles on many of the points above.

Software and code

Policy information about availability of computer code

Data collection	Datasets from GEO were collected and annotated. Summary statistics were computed in R.
Data analysis	A meta-analysis was performed using a model-selection approach. The code is available in https://github.com/AshleyLab/motrpac_public_data_analysis

For manuscripts utilizing custom algorithms or software that are central to the research but not yet described in published literature, software must be made available to editors and reviewers. We strongly encourage code deposition in a community repository (e.g. GitHub). See the Nature Research guidelines for submitting code & software for further information.

Data

Policy information about availability of data

All manuscripts must include a data availability statement. This statement should provide the following information, where applicable:

- Accession codes, unique identifiers, or web links for publicly available datasets
- A list of figures that have associated raw data
- A description of any restrictions on data availability

The curated database (ExTraMeta) generated in this study is available at www.extrameta.org.
Data supporting the findings of this study are available within the article and its supplementary information files. Source data for the meta-analysis are provided with this paper. All computed summary statistics are available here: https://github.com/AshleyLab/motrpac_public_data_analysis

Field-specific reporting

Please select the one below that is the best fit for your research. If you are not sure, read the appropriate sections before making your selection.

- [x] Life sciences
- [ ] Behavioural & social sciences
- [ ] Ecological, evolutionary & environmental sciences

For a reference copy of the document with all sections, see nature.com/documents/nr-reporting-summary-flat.pdf

Life sciences study design

All studies must disclose on these points even when the disclosure is negative.

Sample size	We collected and annotated data from 43 human exercise training studies, including 1,724 samples from human blood or skeletal muscle from 739 subjects in total.
Data exclusions	data from adipose tissue were excluded due to insufficient sample size. Data that were not from gene expression datasets were excluded from the meta-analyses as only gene expression datasets were available in sufficient numbers to conduct a meta-analysis.
Replication	A separate acute endurance exercise cohort of 16 individuals, 8 males and 8 females, was included to validate some of the meta-analysis findings.
Randomization	not applicable for this study as data was gathered from already conducted and published studies
Blinding	not applicable for this study as all analyses are conducted in an unbiased way on already collected datasets

Reporting for specific materials, systems and methods

We require information from authors about some types of materials, experimental systems and methods used in many studies. Here, indicate whether each material, system or method listed is relevant to your study. If you are not sure if a list item applies to your research, read the appropriate section before selecting a response.

Materials & experimental systems

n/a	Involved in the study	
x		Antibodies
x		Eukaryotic cell lines
x		Palaeontology and archaeology
x		Animals and other organisms
	x	Human research participants
x		Clinical data
x		Dual use research of concern

Methods

n/a	Involved in the study	
x		ChIP-seq
x		Flow cytometry
x		MRI-based neuroimaging

Human research participants

Policy information about studies involving human research participants

Population characteristics	The human participants for the validation cohort were young (average age 25), healthy volunteers, 50% males and 50% females. No genotype information was acquired and no clinical information about the subjects is provided in this study.
Recruitment	Participants were recruited through local advertising. Self-selection bias for willingness to provide skeletal muscle samples is present, but unlikely to affect the results as the current analyses are based on intraindividual comparisons.
Ethics oversight	The study was approved by the Regional Ethical Review Board in Stockholm, Sweden

Note that full information on the approval of the study protocol must also be provided in the manuscript.
